# Patients’ Fears and Perceptions Associated with Anesthesia

**DOI:** 10.3390/medicina58111577

**Published:** 2022-11-01

**Authors:** Ksenija Jovanovic, Nevena Kalezic, Sandra Sipetic Grujicic, Vladan Zivaljevic, Milan Jovanovic, Milica Savic, Ranko Trailovic, Milica Vjestica Mrdak, Maja Novovic, Jelena Marinkovic, Biljana Kukic, Tijana Dimkic Tomic, Slobodan Cvetkovic, Lazar Davidovic

**Affiliations:** 1 Center for Anesthesiology and Resuscitation, University Clinical Center of Serbia, 11000 Belgrade, Serbia; 2 Faculty of Medicine, University of Belgrade, Dr Subotica 8, 11000 Belgrade, Serbia; 3 Institute of Epidemiology, Faculty of Medicine, University of Belgrade, 11000 Belgrade, Serbia; 4 Clinic for Endocrine Surgery, University Clinical Center of Serbia, 11000 Belgrade, Serbia; 5 Clinic for Rehabilitation “Dr Miroslav Zotovic”, 11000 Belgrade, Serbia; 6 Clinic for Vascular and Endovascular Surgery, University Clinical Center of Serbia, 11000 Belgrade, Serbia

**Keywords:** fear of anesthesia, anesthesia knowledge, patients’ perceptions, anesthesiologists’ role

## Abstract

*Background and Objectives:* It has been suggested that intense feelings of fear/anxiety and significant patient concerns may affect the perioperative course. Those findings emphasize the importance of surgical patients’ preoperative feelings. Still, current knowledge in this area is based on a limited number of studies. Thus, we think that there is a need to further explore patients’ preoperative fears, better characterize risk factors and reasons for their occurrence, and evaluate patients’ perspectives associated with anesthesia. *Materials and Methods:* A total of 385 patients undergoing vascular surgery were preoperatively interviewed using a questionnaire that included demographics and questions related to patients’ fears and perceptions of anesthesia. Statistical analyses included descriptive statistics, Pearson’s χ^2^ and McNemar tests, and multivariate ordinal logistic regression. *Results:* The main causes of patients’ preoperative fear were surgery (53.2%), potential complications (46.5%), and anesthesia (40%). Female sex was a predictor of surgery and anesthesia-related fear (OR = 3.07, *p* = 0.001; OR = 2.4, *p* = 0.001, respectively). Previous experience lowered the fear of current surgery (OR = 0.65, *p* = 0.031) and anesthesia (OR = 0.6, *p* = 0.017). Type of surgery, type of anesthesia, educational and socioeconomic status, and personal knowledge of an anesthesiologist affected specific anesthesia-related fears. Over 25% of patients did not know that an anesthesiologist is a physician, and only 17.7% knew where anesthesiologists work. Level of education and place of residence influenced patients’ perceptions of anesthesia. *Conclusions:* Anesthesia-related fears are affected by the type of surgery/anesthesia, experience with previous surgery, and personal knowledge of an anesthesiologist. Women, patients with lower education levels, and patients with poorer socioeconomic status are at higher risk of developing those fears. The perception of anesthesiologists is inadequate, and knowledge of anesthesia is poor. Promotion of patient education regarding anesthesia is needed to alleviate those fears and increase understanding of anesthesia.

## 1. Introduction

Anesthesiologists’ contribution to patients’ well-being during surgery is unquestionable. They also play a major role in the preoperative clinics, intensive care units, trauma centers, resuscitation teams, pain clinics, etc. Still, anesthesiology has stayed in the shadows for decades, and the efforts of those medical professionals are not properly recognized, even today. The most probable reason for the underestimation of the anesthesiologist’s importance might be a lack of information and basic knowledge regarding anesthesia in the general population [1]. This, in turn, may contribute to the negative, unfavorable, and erroneous attitudes toward anesthesiology that surgical patients seem to have [2,3,4]. Such opinions may lead to the occurrence of preoperative anxiety and anesthesia-related fear. In small amounts, those feelings are considered a typical physiologic reaction in patients awaiting surgical treatment. However, if those feelings prevail, various complications may arise—even the outcome of surgical treatment may be compromised [1,2,3]. This emphasizes the importance of the general population’s, and especially surgical patients’, perceptions of anesthesia. Although several studies have investigated this topic, data describing experiences and attitudes toward anesthesia in the Serbian population are lacking. Thus, the aim of the present study was to identify the main causes of anesthesia-related fear, the factors associated with those feelings, and to assess patients’ knowledge and perceptions of anesthesia.

## 2. Materials and Methods

### 2.1. Participants

The present study was conducted at the Clinic for Vascular and Endovascular Surgery, University Clinical Center of Serbia, from February until October 2019. After a thorough explanation of the purpose of the study by one of six doctors from the Anesthesia Department, all of the participants voluntarily signed informed consent to participate.

Consecutive patients electively operated on due to aortic, carotid, and peripheral artery disease, under regional and general anesthesia, at our institution were enrolled in the study. Patients who had already undergone some kind of vascular surgical procedure, patients with previously diagnosed anxiety disorders, those with communication problems, and those who declined to participate were excluded.

### 2.2. Data Collection

Data were collected one day before the scheduled procedure, prior to the preoperative anesthetic visit, using a questionnaire specifically created for the purpose of the study. A group of experts developed the questionnaire following a thorough literature review and based on questionnaires used in similar previously published studies [5,6,7]. The questionnaire consisted of three sections. The first section included several questions related to demographics: sex, age, educational background, socioeconomic and employment status, number of household members, place of residence, as well as data on previous surgery and anesthesia. Patients were also asked if they personally knew any anesthesiologist. The second part of the questionnaire had 15 fixed questions for the assessment of surgery- and anesthesia-related fears. Patients were asked to mark the level of each specific fear on a 5-point Likert scale, with 1 being “not at all” and 5 being “extremely”. The last section included 12 fixed questions that referred to the basic knowledge of anesthesia and the importance of anesthesiologists. Data regarding the type of current procedure and anesthetic technique were subsequently obtained from the database implemented in daily practice.

### 2.3. Statistical Analyses

Methods of descriptive statistics were used for the analysis of patient demographics. Continuous variables are presented as mean values ± standard deviation (SD), and categorical variables are shown as absolute numbers (n) with percentages (%). Pearson’s χ^2^ test and McNemar test were used to compare the differences in the frequency of categorical variables. Multivariate ordinal logistic regression was used to model the effects of the patient’s demographic and clinical characteristics (sex, age, education, socioeconomic and employment status, number of household members, place of residence, type of current surgery and anesthesia, history of surgery and anesthesia, and personal knowledge of an anesthesiologist) on specific fears. For every question about fear, a separate multivariate logistic regression analysis was performed. Statistics were performed using SPSS 22.0 (Chicago, IL, USA) and the significance level was set at <0.05.

## 3. Results

Out of 402 questionnaires distributed, 385 valid questionnaires were collected. Sixteen patients were excluded according to the exclusion criteria: five previously had vascular surgery, eleven had an anxiety disorder diagnosed preoperatively, and one patient declined to participate. The mean age of participants was 67.1 ± 7.4 years (range 39 to 86 years) and nearly 80% of them belonged to the male sex. The majority of patients had a primary or high school diploma (74.3%) and were unemployed (81.6%). Almost half of the participants stated that they had fair socioeconomic conditions (46.8%), and patients predominantly lived in a city (65.5%) and in a multimember household (82.6%). Over 60% of patients had already experienced surgery and anesthesia. Nearly 22% of the study participants personally knew an anesthesiologist. Data regarding the current procedure and anesthesia types, as well as basic demographics, are presented in Table 1 and Table S1.

When specific surgery- and anesthesia-related fears were analyzed, on a scale from one to five, patients mostly answered that they were not afraid. The main causes of fear in our study group were surgery (in 53.2% of patients), complications of surgery (in 46.5%), anesthesia (in 40%), postoperative pain (in 36.9%), nausea and vomiting (in 36.9%), stroke (in 33.2%), and not waking up following surgery (31.9%). Additionally, over 10% of patients stated that they were extremely afraid of anesthesia, not waking up following surgery, heart attack, stroke, and complications of surgery (Table 2), Table S2.

According to the results of multivariate logistic regression analysis, age was not associated with any of examined fears. Still, when compared according to sex, significant differences were observed. Women patients expressed a significantly higher level of fear related to surgery, anesthesia, postoperative pain, nausea and vomiting, waking up during, not waking up following surgery, the anesthesiologist’s absence during surgery, memory loss, heart attack, stroke, and complications of surgery. Patients with lower education level were significantly more afraid of the anesthesiologist’s absence during surgery, while those with poorer socioeconomic status showed a significantly higher level of fear regarding not waking up following surgery and having a heart attack during surgery. Patients who were undergoing aortic and carotid surgery were significantly more afraid of a heart attack during surgery; furthermore, those who were undergoing carotid surgery had a greater fear of stroke. The type of anesthesia used was associated with the fear of heart attack; patients having surgery under general anesthesia showed a higher level of fear of this complication. Patients who had already had some kind of surgical intervention showed a lower level of fear of surgery, anesthesia, drowsiness, and memory loss following surgery. Personal knowledge of an anesthesiologist was associated with a lower level of fear of anesthesia and heart attack during anesthesia (Table S1).

Over 25% of patients did not know that an anesthesiologist is a medical doctor, while only 17.7% of respondents knew the exact place of work of an anesthesiologist. Although a majority of patients knew that an anesthesiologist is the one who puts a patient to sleep for surgery, over 40% of patients stated that a surgeon or a nurse wakes up the patient at the end of the surgery. Slightly less than half of the study participants did not know that an anesthesiologist is responsible for monitoring a patient’s vitals during surgery. More than 50% of patients knew that an anesthesiologist takes care of the patient during the whole surgical procedure, but only 2.1% of them knew that anesthesiologists also monitor the patient in the intensive care unit. One-third of the respondents stated that an anesthesiologist and a nurse anesthetist are the same. Thirty percent of patients felt that a nurse anesthetist does not need to be specially trained. When it comes to the role of anesthesiologists, nearly 17% of patients responded that anesthesiologists do not influence the outcome of the surgery. Almost three-quarters of patients with a history of surgery knew the name of the surgeon who performed their previous surgery, while only 6.25% of them knew the name of the anesthesiologist who was in charge of anesthesia (Table 3).

When percentages of correct answers to the questions about anesthesia were com-pared according to sex, no statistically significant differences were noted, except for the question “Who wakes the patient up at the end of the operation?” Statistically, men subjects were significantly more aware that an anesthesiologist wakes up the patient (*p* = 0.012). Additionally, there were no statistically significant differences in the percentage of correct answers about the knowledge of anesthesia in relation to the age of the subjects. Still, significant differences were noticed when correct answers were compared between patients with less than 8 years of education (mandatory education in our country) and those who had completed more than primary school. Patients with more education gave a significantly higher percentage of correct answers to the questions regarding the anesthesiologist’s workplace (*p* = 0.042), who puts the patient to sleep (*p* = 0.001), who wakes the patient up at the end of the surgery (*p* = 0.001), monitoring of vital organs during surgery (*p* = 0.001), the difference between an anesthesiologist and a nurse anesthetist (*p* = 0.001), and to what extent the anesthesiologist influences surgical treatment outcome (*p* = 0.001). Differences in answers to the same questions were also noted when patients were compared according to their place of residence (urban vs. rural areas). Patients who had had previous surgeries gave a significantly higher percentage of correct answers to questions “Who wakes the patient up at the end of the operation?” (*p* = 0.006) and “Who monitors the functioning of vital organs during surgery?” (*p* = 0.04) (Table S2).

## 4. Discussion

The association between anesthesia and fear/anxiety has been documented for almost 50 years: in 1972, Ramsay found that anesthesia was the main reason for fear in patients waiting for surgical treatment [8]. More recent evidence suggests that there is a wide range of preoperative patient fears [9,10,11,12,13]. Despite the fact that it can alter anesthetic management during surgery and even complicate postoperative course [14,15,16], current knowledge of preoperative (anesthesia-related) fears, risk factors, and reasons for their occurrence is based on very limited data. Simultaneously, patients’ perceptions regarding anesthesia do not seem to have been sufficiently studied. Therefore, the aim of the current study was to broaden current knowledge in this area. Our results are significant in at least three major respects.

First of all, the present study shows that a significant percentage of surgical patients (40%) experience some degree of fear related exclusively to anesthesia. Unlike surgery, anesthesia may seem abstract to surgical patients, and the majority of them describe anesthesia-related fear as the fear of the unknown [17]. Defined by Carleton in 2016 as “an individual’s propensity to experience fear caused by the perceived absence of information at any level of consciousness or point of processing” [18], this type of fear probably results from insufficient knowledge and unanswered questions regarding the basic principles of anesthesia [19]. Based on our results, anesthesia is the third most common cause of patients’ preoperative fears, following surgery and surgical complications. These findings differ from previous results reported in the literature. The percentage of patients who are afraid of anesthesia is even greater—varying from 76 to 81 percent [5,20]. The fact that our participants underwent surgery of the aortic, carotid, or peripheral arteries, which are marked as high-risk procedures [21], might clarify why surgery seemed more frightening than anesthesia to the patients in our study.

Secondly, the present results show that women are more prone to experience fear during the preoperative period. Namely, female sex was a predictor for a higher level of fear in 11 out of 15 questions, and women were threefold more likely to be afraid of surgery and 2.4 fold more likely to be afraid of anesthesia than men. Those findings confirm the results of previously published studies, which state that women are more likely to experience fears related to anesthesia two to five times more often than men [5,10,22]. According to Mavridou et al. [5], those sex-related differences can be explained by the fact that men are less likely to admit and express their fears due to differences in social standards.

In addition to sex, the level of education and socioeconomic status also affected the occurrence of preoperative fears related to anesthesia in our study, but to a much lesser extent. Although minor in the present study, the influence of those patients’ characteristics should not be neglected because the literature data also show that the degree of fear increases with lower educational levels [23] and poorer socioeconomic conditions [24]. Furthermore, it was reported that the level of fear is dependent on the severity of surgery [25] and that certain anesthesia techniques might contribute to the development of a higher level of preoperative fear in patients awaiting surgical treatment [23]. Our study provides further evidence to support this association.

While age was designated as a risk factor for the development of preoperative anesthesia-related fear in a significant number of studies [5,10,26], with younger patients tending to have a higher level of anesthesia-related fears, no significant differences were observed according to age in our study. The potential reason behind this discrepancy can be found in the characteristics of our study sample: our patients were notably older than those in the above-mentioned studies. On the other hand, we found that personal knowledge of an anesthesiologist and a history of surgery/anesthesia lowered the incidence of preoperative fears. We can assume that those patients were less afraid because they probably knew what to expect from their previous experience or were more informed about anesthesia. The results of the study by Fitzgerald and Elder also support this assumption: informational handouts of only one typewritten page decreased patients’ fears of anesthesia and surgery by as much as 40% [27]. This justifies the need for the education of patients and the promotion of anesthesia among the general population.

Finally, we demonstrated that the general knowledge of surgical patients regarding anesthesia is inadequate and that anesthesia is underappreciated. Even though the majority of our patients (slightly above 73%) were aware of anesthesiologists’ qualifications, anesthesiologists’ responsibilities and roles outside the operating theater, their substantial influence on patients’ well-being during the whole perioperative period, as well as the main scope of anesthesia practice remain unknown. Our results agree with the results of many similar studies [28,29,30]. Unfortunately, similar patient perceptions regarding anesthesia were described 26 years ago [31], suggesting that anesthesiologists have not improved their image in the general population. Those perceptions not only affect the general population and surgical patients, but medical students as well. In some countries, it has been shown that only 1% of medical school graduates choose anesthesiology as their future specialty [32,33,34]. This low level of interest in anesthesia may directly jeopardize the future of the anesthesiologic practice. Promotion of anesthesia among the general population and medical students, along with the improvement in working conditions, should change the general population’s attitude toward anesthesiology.

The level of education and place of residence had a significant impact on anesthesia knowledge and patients’ perceptions regarding the importance of anesthesiologists in our study. More educated patients and those living in urban areas answered significantly more questions correctly. These patients probably had better availability of information regarding anesthesia. Education level was also a predictor of patients’ increased knowledge of anesthesia in the studies of other authors [29,35]. On the other hand, past surgery and anesthesia experience had only a minor impact on the percentage of correct answers in our study. We can assume that those patients were not properly informed about the fundamental principles of anesthesia at their prior surgery. Sex and age did not affect our patient’s answers at all, probably due to the typical characteristics of vascular patients (mostly male sex and of older age). Those results are inconsistent with the findings of some authors: younger age and previous experience with surgery imply better knowledge regarding anesthesia [30,36].

## 5. Strengths and Limitations

The present study has several limitations. First of all, an observational, single-center design, and a homogenous sample limit the generalization of our results. A significant percentage of our patients already had previous experience with surgery and anesthesia, which may have affected some of the examined issues. Additionally, the present study was conducted during the pre-COVID era. Hopefully, future studies will prove that at least some of the patients’ beliefs regarding anesthesiologists have changed. Still, the present study included a relatively large sample of patients prepared for different types of anesthesia in a specific setting, so it represents a solid basis for future research.

## 6. Conclusions

Anesthesia-related fear is present in a significant percentage of surgical patients. Type of surgery/anesthesia, previous experience with surgery and anesthesia, and personal knowledge of an anesthesiologist are factors that may influence the occurrence of fear related to anesthesia and surgery. Women, patients with lower education levels, and patients with poorer socioeconomic status are at higher risk of developing preoperative fears. Patients have poor knowledge and perceptions regarding anesthesia and the role of anesthesiologists. A higher level of education and urban area of residence improve those perceptions.

Surgical patients experience anesthesia-related fears, and those fears may arise as a direct consequence of poor knowledge. In order to reduce the incidence of those fears, raise the general population’s understanding of anesthesia, and improve anesthetists’ reputations, promotion of patient education and anesthesia are needed. Health systems, along with national societies, should make substantial efforts to organize campaigns on social networks, interviews with professionals and patients on national broadcast channels, and street posters, focusing their efforts toward women, patients from rural areas, and less educated patients. The ultimate goals should be the alleviation of patients’ fears, reduction in healthcare costs, and accessibility of anesthesia information to the public.

## Figures and Tables

**Table 1:** Basic demographic and clinical characteristics of patients.

Characteristics	Number (%)
**Age**	
39–67 years	197 (51.2%)
67–86 years	188 (49.8%)
**Sex**	
Female	80 (20.8%)
Male	305 (79.2%)
**Education**	
Not literate/Incomplete primary school	15 (3.9%)
Primary	72 (18.7%)
High school	214 (55.6%)
University degree/Master’s/Ph.D.	84 (21.8%)
**Socioeconomic status**	
Good	156 (40.5%)
Fair	180 (46.8%)
Bad	49 (12.7%)
**Employment status**	
Unemployed	314 (81.6%)
Employed	71 (18.4%)
**Number of household members**	
Lives alone	67 (17.4%)
Multimember household	318 (82.6%)
**Place of residence**	
City	252 (65.5%)
Province	133 (34.5%)
**Surgery type**	
Peripheral arteries	85 (22.1%)
Carotid	157 (40.8%)
Aortic	143 (37.1%)
**Type of anesthesia**	
General	160 (41.6%)
Regional	225 (58.4%)
**Previous surgery and anesthesia**	
No	145 (37.7%)
Yes	240 (62.3%)
**Do you personally know any anesthesiologist?**	
No	302 (78.4%)
Yes	83 (21.6%)

**Table 2:** Questions for the assessment of specific fears.

	Percentages (%)				
*Please mark your answer on a scale from 1 to 5,* *with 1 being “not at all” and 5 being “extremely”*	1	2	3	4	5
1. How much are you afraid of surgery?	46.8	18.4	18.7	7.5	8.6
2. How much are you afraid of anesthesia?	60.0	11.2	11.9	6.8	10.1
3. Are you afraid of needles and injections needed for anesthesia?	74.8	8.3	6.0	1.8	9.1
4. Are you afraid of pain following the surgery?	63.1	15.1	11.4	4.4	6.0
5. Are you afraid of nausea and vomiting following the surgery?	63.1	15.1	11.4	4.4	6.0
6. Are you afraid of headache following the surgery?	79.2	8.8	5.5	2.3	4.2
7. Are you afraid that you will be drowsy following surgery?	85.7	5.7	3.6	3.4	1.6
8. Are you afraid that you will wake up during surgery?	78.7	5.7	5.7	3.1	6.8
9. Are you afraid that you will talk in your sleep during surgery?	89.6	2.9	3.4	1.8	2.3
10. Are you afraid that you will not wake up after surgery?	68.1	8.1	7.8	2.9	13.2
11. Are you afraid that the anesthesiologist will leave the room during surgery?	89.9	3.1	3.6	1.0	2.3
12. Are you afraid of memory loss due to anesthesia?	76.1	7.8	4.7	2.1	9.4
13. Are you afraid of a heart attack during anesthesia?	72.2	7.5	5.7	3.9	10.6
14. Are you afraid of stroke during anesthesia?	66.8	10.1	5.7	3.9	13.5
15. Are you afraid of complications of the surgery?	53.5	19.2	11.9	4.2	11.2

**Table 3:** Patients’ knowledge of anesthesia and perceptions of anesthesiologists’ role.

Question	Number	Percentage
1. Must anesthesiologists have a medical degree?		
(a) yes	282	73.30%
(b) no	89	23.10%
(c) I don’t know	14	3.60%
2. Where does the anesthesiologist work?		
(a) in the operating room	287	74.60%
(b) in the intensive care unit	12	3.10%
(c) in the resuscitation unit	4	1%
(d) all of the above	68	17.70%
(e) none of the above	4	1%
(f) I don’t know	10	2.60%
3. Who puts the patient to sleep for surgery?		
(a) surgeon	12	3.10%
(b) nurse	31	8%
(c) anesthesiologist	339	88.10%
(d) I don’t know	3	0.80%
4. Who wakes the patient up at the end of the operation?		
(a) surgeon	44	11.40%
(b) nurse	118	30.60%
(c) anesthesiologist	207	53.80%
(d) I don’t know	16	4.20%
5. Who monitors the functioning of vital organs during surgery?		
(a) surgeon	108	28.10%
(b) nurse	51	13.20%
(c) anesthesiologist	209	54.30%
(d) I don’t know	17	4.40%
6. At what stages of surgery does the anesthesiologist take care of the patient?		
(a) at the beginning	68	17.70%
(b) at the end	8	2.10%
(c) throughout the surgery	213	55.30%
(d) in the intensive care unit	8	2.10%
(e) all of the above	78	20.20%
(f) none of the above	5	1.30%
(g) I don’t know	5	1.30%
7. Are anesthesiologists and nurse anesthetists the same?		
(a) yes	134	34.80%
(b) no	171	44.40%
(c) I don’t know	80	20.80%
8. Does the nurse anesthetist have to be specially trained?		
(a) no	118	30.60%
(b) yes	246	63.90%
(c) I don’t know	21	5.50%
9. To what extent does an anesthesiologist influence the outcome of surgery?		
(a) does not affect at all	65	16.90%
(b) affects, but less than the surgeon	90	23.40%
(c) affects as much as the surgeon	188	48.80%
(d) affects more than the surgeon	35	9.10%
(e) I don’t know	7	1.80%
10. Do you remember the name of the surgeon who operated on you the previous time?		
(a) yes	179	46.50%
(b) no	61	15.80%
(c) I have not had surgery so far	145	37.70%
11. Do you remember the name of any nurse from the surgical ward where you were hospitalized due to a previous operation?		
(a) yes	63	16.40%
(b) no	177	46%
(c) I have not had surgery so far	145	37.60%
12. Do you remember the name of the anesthesiologist in charge of anesthesia for your previous surgery?		
(a) yes	15	3.90%
(b) no	225	58.40%
(c) I have not had surgery so far	145	37.70%

## Data Availability

The data that support the findings of this study are available from the corresponding author upon reasonable request.

## Supplementary Materials

The following supporting information can be downloaded at: https://www.mdpi.com/article/10.3390/medicina58111577/s1, Table S1: Results of multivariate logistic regression analysis: specific fears in relation to patients’ characteristics; Table S2: Percentage of correct answers to the questions regarding knowledge of anesthesia and differences regarding sex, age, education level, place of residence, and previous surgery.
