# Supplementary material for: Patients’ Fears and Perceptions Associated with Anesthesia
Source: Medicina (Kaunas). 2022 Nov 1;58(11):1577. doi: 10.3390/medicina58111577 (PMC9693447; doi:10.3390/medicina58111577)
Supplement: Supplementary file 1 [file medicina-58-01577-s001.zip › Table S1.pdf]

Table S1. Results of multivariate logistic regression analysis: specific fears in relation to patients' characteristics.

Question	1	2	3	4	5	6	7	8	9	10	11	12	13	14	15
Gender (female/male)	**3.07 (1.84-5.1) p=0.001***	**2.4 (1.4-4.1) p=0.001***	1.75 (0.91-3.18) p=0.065	**2.59 (1.51-4.45) p=0.001***	**2.39 (1.24-4.59) p=0.009***	1.84 (0.98-3.47) p=0.057	1.69 (0.8-3.57) p=0.173	**2.45 (1.3-4.61) p=0.006***	2.05 (0.87-4.79) p=0.099	**2.46 (1.38-4.37) p=0.002***	**3.37 (1.45-7.88) p=0.005***	**1.95 (1.07-3.54) p=0.028***	**2.16 (1.21-3.85) p=0.01***	**2.41 (1.4-4.15) p=0.002***	**2.61 (1.56-4.38) p=0.001***
Level of education (1. not literate/incomplete primary school; 2. primary school; 3. high school; 4. University degree/Master's/PhD)	1,31 (0,99 - 1,74) p=0,056	1,14 (0,85-1,54) p=0,382	1,07 (0,76-1,51) p=0,712	1,34 (0,99-1,82) p=0,062	0,94 (0,64-1,37) p=0,729	0,99 (0,69-1,43) p=0,974	1,31 (0,85-2,01) p=0,222	0,95 (0,66-1,38) p=0,796	1,1 (0,66-1,81) p=0,72	1,08 (0,78-1,49) p=0,649	**1,79 (1,06-3) p=0,028**	0,89 (0,63-1,24) p=0,483	0,92 (0,66-1,27) p=0,611	0,88 (0,65-1,2) p=0,43	0,99 (0,75-1,31) p=0,945
Socioeconomic status (1.bad; 2. fair; 3.good)	1,09 (0,81-1,45) p=0,577	1,16 (0,85-1,58) p=0,343	1,2 (0,84-1,71) p=0,307	0,86 (0,62-1,18) p=0,344	0,9 (0,61-1,35) p=0,729	1,04 (0,71-1,51) p=0,852	0,74 (0,47-1,16) p=0,184	1,27 (0,87-1,84) p=0,219	1,01 (0,61-1,67) p=0,978	**1,55 (1,11-2,15) p=0,009**	1,47 (0,87-2,49) p=0,146	1,328 (0,92-1,89) p=0,131	**1,47 (1,04-2,07) p=0,03**	1,25 (0,9-1,73) p=0,181	1,26 (0,94-1,7) p=0,129
Type of surgery (peripheral arteries /carotid/aortic)	1.78 (0.69-4.55) p=0.23	1.76 (0.65-4.77) p=0.265	0.437 (0.48-5.35) p=0.437	1.67 (0.63-4.42) p=0.303	0.24 (0.03-1.9) p=0.176	0.56 (0.12-2.69) p=0.467	0.96 (0.21-4.51) p=0.862	0.77 (0.48-1.23) p=0.272	2.05 (0.52-8.16) p=0.308	0.91 (0.3-2.75) p=0.871	**5 (1.28-20.53) p=0.026*** [a]	2.67 (0.85-8.41) p=0.092	**4.44 (1.59-12.4) p=0.004*** [b]	**3.8 (1.18-12.23) p=0.025*** [c]	1.76 (0.67-4.61) p=0.248
Type of anesthesia (general/regional)	1.72 (0.66-4.48) p=0.162	1.79 (0.65-4.9) p=0.259	0.93 (0.28-3.09) p=0.91	1.69 (0.89-3.2) p=0.108	0.21 (0.03-2.15) p=0.147	0.5 (0.1-2.41) p=0.386	0.77 (0.17-3.55) p=0.737	1.56 (0.49-4.99) p=0.451	2.72 (0.64-11.46) p=0.44	1.89 (0.95-3.75) p=0.069	3.3 (0.85-12.84) p=0.084	1.96 (0.63-6.14) p=0.246	**5.63 (1.96-16.17) p=0.001***	2.38 (0.82-6.88) p=0.11	1.76 (0.66-4.68) p=0.257
Previous surgery and anesthesia (yes/no)	**0.65 (0.44-0.96) p=0.031***	**0.6 (0.4-0.91) p=0.017***	0.65 (0.4-1.05) p=0.079	0.93 (0.61-1.44) p=0.759	0.87 (0.5-1.49) p=0.603	0.73 (0.44-1.22) p=0.226	**0.48 (0.26-0.88) p=0.018***	0.82 (0.49-1.38) p=0.458	0.82 (0.41-1.62) p=0.564	0.98 (0.62-1.54) p=0.923	0.81 (0.4-1.67) p=0.571	**0.61 (0.37-1) p=0.048***	0.77 (0.48-1.23) p=0.272	0.64 (0.41-1) p=0.052	0.69 (0.46-1.04) p=0.075
Personal knowledge of an anesthesiologist (no/yes)	1.41 (0.87-2.28) p=0.162	**2.53 (1.43-4.48) p=0.001***	1.33 (0.71-2.49) p=0.377	1.06 (0.64-1.76) p=0.828	1.1 (0.56-2.15) p=0.788	1.38 (0.7-2.69) p=0.351	1.89 (0.79-4.53) p=0.153	1.98 (0.95-4.11) p=0.068	2.85 (0.95-8.53) p=0.061	1.47 (0.82-2.61) p=0.192	2.38 (0.82-6.9) p=0.112	1.41 (0.73-2.76) p=0.309	**2.57 (1.28-5.15) p=0.001***	1.47 (0.82-2.64) p=0.195	1.6 (0.96-2.65) p=0.072

Legend: odds ratio, 95% confidence interval, * statistically significant, [a] peripheral arteries, [b] carotid and aortic, [c] carotid.
