# Supplementary material for: Patients’ Fears and Perceptions Associated with Anesthesia
Source: Medicina (Kaunas). 2022 Nov 1;58(11):1577. doi: 10.3390/medicina58111577 (PMC9693447; doi:10.3390/medicina58111577)
Supplement: Supplementary file 1 [file medicina-58-01577-s001.zip › Table S2.pdf]

Table S2. Percentages of correct answers to the questions regarding knowledge of anesthesia and differences regarding gender, age, education level, place of residence, and previous surgery.

Question	Gender			Age (years)			Education (years)			Place of residence			Previous surgery		
	male	female	*p*	≤67	>67	*p*	≤8	>8	*p*	city	province	*p*	no	yes	*p*
1. Do anesthesiologists must have a medical degree?	73.8	71.2	0.65	71.6	75	0.448	69	74.5	0.305	75.8	68.4	0.12	69.7	75.4	0.216
2. Where does the anesthesiologist work?	18	16.2	0.71	18.3	17	0.747	10.3	19.8	**0.042**	21.8	9.8	**0.003**	16.6	18.3	0.657
3. Who puts the patient to sleep for surgery?	87.9	88.8	0.829	89.3	86.7	0.425	74.7	91.9	**0.001**	91.7	81.2	**0.003**	87.6	88.3	0.827
4. Who wakes the patient up at the end of the operation?	57	41.2	**0.012**	53.8	53.7	0.987	31	60.4	**0.001**	57.5	46.6	**0.041**	44.8	59.2	**0.006**
5. Who monitors the functioning of vital organs during surgery?	54.1	55	0.885	58.4	50	0.099	39.1	58.7	**0.001**	58.7	45.9	**0.016**	47.6	58.3	**0.04**
6. At what stages of surgery does the anesthesiologist take care of the patient?	19.7	22.5	0.575	20.8	19.7	0.782	13.8	22.1	0.088	23	15	0.064	20.7	20	0.807
7. Are anesthesiologist and nurse anesthetist the same?	46.6	36.2	0.099	47.7	41	0.182	28.7	49	**0.001**	49.2	35.3	**0.009**	42.8	45.4	0.611
8. Does the nurse anesthetist have to be specially trained?	63.6	65	0.817	62.4	65.4	0.542	52.9	67.1	**0.015**	69.8	52.6	**0.001**	60.7	65.8	0.309
9. To what extent does an anesthesiologist influence the outcome of surgery?	50.5	42.5	0.203	51.8	45.7	0.237	32.2	53.7	**0.001**	53.6	39.8	**0.01**	46.2	50.4	0.423
